# Can the health related quality of life measure QOLIBRI- overall scale (OS) be of use after stroke? A validation study

**DOI:** 10.1186/s12883-018-1101-9

**Published:** 2018-07-18

**Authors:** Guri Heiberg, Synne Garder Pedersen, Oddgeir Friborg, Jørgen Feldbæk Nielsen, Henriette Stabel Holm, Nicole Steinbüchel von, Cathrine Arntzen, Audny Anke

**Affiliations:** 1 Department of Rehabilitation, University Hospital of North Norway, Tromsø, Norway; 2 Department of Clinical Medicine, Faculty of Health Sciences, University of Tromsø, The Arctic University of Norway, Tromsø, Norway; 3 Department of Health and Care Sciences, Faculty of Health Sciences, University of Tromsø The Artic University of Norway, Tromsø, Norway; 4 Department of Psychology, Faculty of Health Sciences, University of Tromsø, the Artic University of Norway, Tromsø, Norway; 5 Hammel Neurorehabilitation Centre and University Research Clinic, Aarhus University, Aarhus, Denmark; 6 Universitäty Medicine, Georg-August-University, Göttingen, Germany; 7 Department of Rehabilitation, University Hospital of North Norway- Harstad, 9480 Harstad, Norway

**Keywords:** QOLIBRI-OS, Stroke, Health related quality of life, Validity

## Abstract

**Background:**

Brief measures of health-related quality of life (HRQOL) that assess both patient-reported functioning and well-being after stroke are scarce. The objective of this study was to examine reliability and validity of one of these measures, the patient-reported Quality of Life after Brain Injury–Overall Scale (QOLIBRI-OS), in patients after stroke.

**Methods:**

Stroke survivors were examined prospectively using survey methods.

Core survey data (*n* = 125) and retest data (*n* = 36) were obtained at 3 and 12 months, respectively. Item properties (distribution, floor and ceiling effects), psychometric properties (reliability and model fit), and validity (correlations with established measures of anxiety, depression and HRQOL) of the QOLIBRI-OS were examined.

**Results:**

Missing responses on the questionnaire were low (0.5%). All items were positively skewed. No floor effects were present, whereas five out of six items showed ceiling effects. The summary QOLIBRI-OS score exhibited no floor or ceiling effects, and had excellent internal consistency (Cronbach’s α =0.93). All item-total correlations were high (0.73–0.88). The test-retest reliability of single items varied from 0.74 to 0.91 and was 0.93 for the overall score. The confirmatory factor analysis yielded an excellent fit for a five-item version and provided tentative support for the original six-item version. The convergent validity correlations were in the hypothesized directions, thus supporting the construct validity.

**Conclusions:**

The brief QOLIBRI-OS is a valid and reliable brief health-related outcome measure that is appropriate for screening HRQOL in patients after stroke.

## Background

Strokes are associated with complex physical, cognitive and psychosocial consequences that pose challenges to valid long-term outcome assessments [1, 2]. Due to a combination of functional, psychological and social constraints, the use of patients reported outcomes (PROs) to assess progress following treatment is advocated [3, 4]. PROs also seek to ascertain patients’ views of the severity of their symptoms and functional status [5].

Generic and disease-specific health related quality of life (HRQOL) instruments assess consequences of health conditions on quality of life comprising psychological, physical, social and daily-life domains [6]. Both generic and disease-specific scales are used following stroke [7–9].

A comprehensive evaluation of the available HRQOL measures found that generic scales had limited value due to their lack of specificity to particular conditions and low responsiveness to change [7]. In the past decade, the use of stroke-specific scales has increased [10].

Stroke-specific HRQOL measures should ideally be reliable, valid, responsive, precise and appropriate as well as feasible, interpretable and easy to complete [3, 11–13]. Examples of these types of measures are the Stroke-Specific Quality of Life (SS-QOL) scale [14] and the Stroke Impact Scale (SIS) [15], which both have shown good psychometric properties and been translated into several languages [16–18]. Although these scales adequately assess functional problems post-stroke, their comprehensive approach, i.e., inclusion of a large number of items covering multiple domains, reduce their feasibility in research and clinical use, especially for patients with cognitive deficits [19] or fatigue post-stroke [20]*.* A brief HRQOL measure could be useful for screening or in situations where the workload should be minimal. Additionally, a brief disease-specific version of the SIS with eight items has been developed [21], but this index does not address satisfaction, subjective functioning and subjective health status. Moreover, to compare conditions between patients with different disorders the measure has to be validated for use in several diagnostic groups.

In literature search of a suitable brief instrument assessing well-being, according to patient-reported satisfaction and important functional domains following stroke, the short Quality of Life after Brain injury Overall Scale (QOLIBRI-OS) [22] was identified as a possible option. This instrument was cross-culturally developed in six European countries between 2000 and 2010, and validated in more than 2000 patients after traumatic brain injury (TBI) [23].

The QOLIBRI-OS is a brief TBI-specific HRQOL index that addresses wellbeing and functioning [22]. The psychometric properties for the QOLIBRI-OS after TBI are satisfactory to good and are highly correlated with the 37 QOLIBRI scale (six subscales), indicating that a comparable construct is assessed [22]. The six items of the QOLIBRI-OS assess overall satisfaction with physical function, cognition, emotional status, ability to perform daily activities, personal life and social relationships, and satisfaction with the current situation and future prospects. A confirmatory factor analysis of the scale seem to support uni-dimensionality; however with some reservations as absolute fit seems clearly poorer (i.e., RMSEA = .07) than the relative fit (e.g., CFI = .98) [22]. QOLIBRI-OS has also been validated for patients with aneurysmal subarachnoid haemorrhage [24].

Stroke has important cognitive, emotional and physical clinical consequences that are similar to those of TBI, even though the health conditions differ in pathogenesis [25, 26]. Thus, the aim of this study was to investigate whether the QOLIBRI-OS is uni-dimensional and a reliable and valid measure of HRQOL post-stroke. To investigate its construct validity, we hypothesized positive correlations between the QOLIBRI-OS and the other HRQOL measures and negative correlations between the QOLIBRI-OS and psychological distress. In addition, concurrent relations of the individual QOLIBRI-items with relevant measures were explored.

## Methods

This validation study is a part of a larger stroke study consecutively enrolling all patients, who were admitted to the stroke units of the University Hospital of Northern Norway (UNN) between March 2014 and December 2014.The inclusion criteria were in accordance with those of the National Stroke Registry. The exclusion criteria were age below 18 years, residence outside the hospital’s region or foreign nationality. Patients with stroke related to brain malignancy, brain trauma or subarachnoid haemorrhage were excluded. A few patients who received acute stroke care in wards other than stroke units, due to the presence of other serious diseases, were also excluded. In total, 161 of 214 eligible patients with ischaemic or haemorrhagic stroke (ICD10 codes I.61 and I.63) consented to participate in the validation study, and 125 finally answered the questionnaire. While the response rate for eligible patients was 56%, the response rate for included consenting patients at 3 months was.

**Fig. 1:**
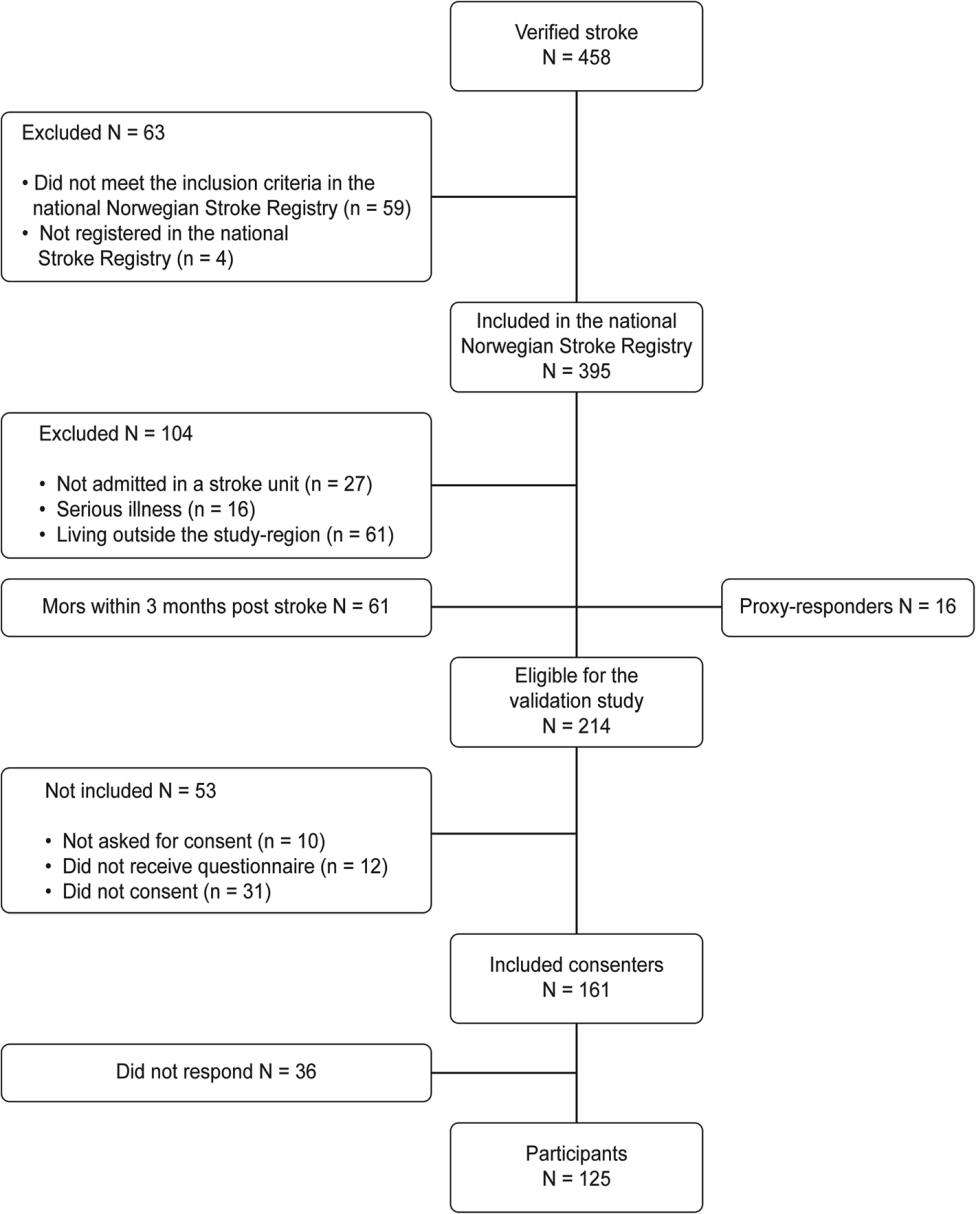
Flowchart of persons with acute ischemic or hemorrhagic stroke registered during the recruiting period

125 /161 = 78%. The flowchart in Fig. 1 shows more information on patient enrolment.

The study was approved by the Norwegian Regional Committee for Medical and Health Research Ethics (2013/ 1472).

### Data collection

Patients were recruited during hospitalization in the stroke unit or by telephone within 3 months of discharge. Participants were asked to provide written consent. A local coordinator at all participating hospitals distributed the questionnaires by mail. Self-reported data were collected 3 months after stroke. Incomplete questionnaires were completed by filling in all missing items after an additional telephone interview. When up to two responses on any questionnaire were missing, mean imputation was performed. Questionnaires with more than 2 missing data points were excluded. Test-retest analysis of the QOLIBRI-OS was performed at 12-month follow-up due to the expected stability in functioning post-stroke [27] at this time point**.** The first 40 participants who answered at 12 months were asked to complete the retest in a 7- to 12-day period. Of these, 36 participants completed and returned the QOLIBRI-OS within the timeframe, which provides a response-rate of 90%. We conducted statistical tests (e.g., Student t- and chi-square tests) comparing the retest group (*n* = 36) with those not retested (*n* = 89), but no significant differences in any demographic characteristics or stroke severity emerged.

### Demographic and stroke registry data

Information about age, gender, living condition and stroke was collected from the Norwegian Stroke Registry. Questions regarding education, marital status and work status were included in the mailed questionnaires, or were collected from the medical records after consent. Function was assessed with the Modified Rankin scale (MRS) [28], a clinician-reported measure of global disability widely used to evaluate post-stroke outcomes [28]. The scale consists of six categories assessing the level of independence, ranging from independent to bedridden or death. There is extensive evidence on the validity of the MRS [28]. In our study, the MRS was registered at 3 months after telephone interviews, as part of the national stroke registry registration.

### Participants

Sociodemographic and clinical characteristics of the 125 participants are shown in Table 1. The average age was 70.5 years, and 62% were male. Approximately 50% of patients had less than 11 years of education, and three out of four had retired before stroke.

**Table 1:** Sociodemographic and stroke characteristics of the participants and non-responders

	Participants *N* = 125	Non-responders *N* = 36	*P*-values
Age at time of stroke, Mean (SD)	70.5 (13.1)	75 (13.6)	< 0.05
Gender, n (%)			
Female	48 (38)	16 (44)	
Male	77 (62)	20 (56)	0.34
Stroke subtype, n (%)			
Ischaemic	113 (90)	31 (86)	
Haemorrhagic	12 (10)	5 (14)	0.33
Marital status at time of stroke, n (%)			
Married/cohabitant	80 (64)	16 (45)	
Widowed/single	45 (36)	20 (55)	< 0.05
Education, time of stroke, n (%)			
≤ 10 years (y)	60 (48)	–	
> 10	62 (50)	–	–
Unknown	3 (2)	–	
Living conditions at 3 months, n (%)			
Home, without assistance	92 (73)	12 (33)	
Home, with assistance	23 (19)	14 (39)	< 0.01^a^
Institution/residence for the elderly	10 (8)	10 (28)	
Work status at 3 months, n (%)			
Student/Unemployed/Working fulltime or part-time	23 (18)	3 (8)	0.77
Retired/ Sick-leave	102 (82)	33 (92)	
MRS at 3 months, n (%)			
0–1 no symptoms or significant disability	84 (67)	15 (42)	
2–3 slight or moderate disability	33 (26)	16 (44)	
4–5 severe disability	8 (7)	7 (14)	< 0.05^b^

^a^Significantly more responders than non-responders lived at home without assistance vs. at home with assistance/in institution at 3 months after stroke

^b^Wilcoxon signed rank test

At 3 months after stroke approximately 75% lived at home without personal assistance. Compared to those who did not respond (*n* = 36), participants were 5 years younger and a larger proportion lived at home at 3 months. The participants and non-responders differed statistically significantly in age, MRS score at 3 months, and proportion living in an institution and in need of assistance. Gender and stroke subtypes were similar in both groups (Table 1).

Comparisons between participants and patients that were eligible for the validation study, but did not participate, were performed only for age and gender for ethical reasons. However, there were no statistically significant differences in these demographic data between the participants and the patients who refused to participate or between those who, due to a administrate failure, were not contacted.

### Measurements

The QOLIBRI-OS comprises six items that assess the degree of overall satisfaction with “Physical Condition”, “Cognition”, “Emotions”, “Ability to Perform Daily Activities”, “Personal and Social Life,” and “Current Situation and Future Prospects”. A Likert scale provides the following five response categories: not at all (score 1), slightly (score 2) moderately (score 3), quite (score 4), very (score 5) for each item [22]. Accordingly, item score range is 1–5 and sum score range 6–30.

Von Steinbuchel et al. [22] arithmetically converted the sum of all items to a percentage scale (0–100). In the present study, both the raw item scores and the overall sum score were used. The QOLIBRI-OS has demonstrated good internal consistency with a Cronbach’s α value of 0.86 in patients after TBI [22] and 0.88 in patients with subarachnoid haemorrhage. [24]. The QOLIBRI full scale (37 items) questionnaire has been examined in a Norwegian study of patients after TBI and showed metric properties supporting the reliability and factor structure. To date, the QOLIBRI-OS (6 items) has not been validated in Norwegian samples. ​ The QOLIBRI-OS was translated into Norwegian in 2008 in accordance with recommended procedures and is used in a longitudinal international observational study (the European Union Study CENTER-TBI-HEALTH. 2013.2.2.1–1). [29, 30] The translation used in our study was slightly modified to improve language fluency, and checked with back translation by a professional translation service. According to a bilingual professional translator the semantic meaning in our Norwegian version express the meaning of the original English version.

The Hospital Anxiety and Depression Scale (HADS), originally published by Zigmond and Snaith in 1983 [31], is a widely used instrument that screens for non-vegetative symptoms of anxiety (seven items) and depression (seven items) [32]. The HADS items are scored from 0 to 3 with higher scores indicating worse symptoms. A cut-off score of 8 indicates a possible diagnosis of anxiety or depression [33]. The total score (HADS-14) can also be used as a global measure of psychological distress [34]. The HADS questionnaire has been applied several times in Norwegian populations [29], also post-stroke [35].

The EuroQol Five Dimensions Questionnaire (EQ-5D) [36] is a three-level generic HRQOL questionnaire comprising 5 items measuring the dimensions of mobility, self-care, ability to perform daily activities, pain/discomfort and anxiety and depression [37]. The levels are rated as 1, 2, or 3, indicating no (1), some (2), and considerable problems (3). Each dimension can be scored separately. The questionnaire includes the EuroQol Visual analogue Scale.

(EQ-VAS), which is a 0–100 visual analogue scale intended to measure actual self-reported health status from worst to best imaginable health [38].

The Stroke Specific Quality of Life (SS-QOL) scale [14] assesses the functional impact of stroke across 12 domains using 49 items and a five-point Likert scale where higher scores indicate better functioning. The SS-QOL measures energy, mood, family roles, language, mobility, self-care, social roles, thinking, personality, and upper extremity function, vision and work/ productivity. A sum score can be extracted from each domain. Separate domain scores are obtained from unweighted average of all items belonging to a particular domain, but the overall SS-QOL score is most often used as the primary outcome. The SS-QOL scale has recently been translated into Norwegian in accordance with recommended procedures [39, 40].

## Validation study design

The construct and criterion-related validity of the QOLIBRI-OS were examined in a confirmatory factor analysis and as concurrent correlations with theoretically related measures, respectively.The instruments chosen represent different aspects like stroke specific health related functions in HRQOL-measures, generic health related quality of life instruments, single questions and instruments assessing anxiety and depression. Moreover, the criterion-related measures included in our study are validated in Norwegian samples.The convergent and divergent validity of the QOLIBRI-OS, as one specific type of criterion-related validity, were supported if the Spearman rank-order correlations with the HADS total and anxiety scales were negative and the EQ-5D and SS-QOL were positive. Such correlations were calculated for both the QOLIBRI-OS total and item scores. The direction of these a priori hypothesised correlations were based on the literature review in the introduction. According to the COSMIN guidelines [41], the overall construct validity is rated positively if the hypothesized relationships are specified in advance and supported in at least 75% of the reported results and based on a minimum of 50 patients.

Correlations above 0.50, between 0.31 and 0.49 and less than 0.30 were considered high, moderate and low, respectively [42]. Based on the literature review, we expected moderate to strong correlations between the QOLIBRI-OS and the criterion measures (see Table 2).The psychometric results from the current study were also used to re-evaluate the content validity of the QOLIBRI-OS, and discuss improvements.

**Table 2:** Construct validity of the QOLIBRI-OS at 3 months after stroke

Items	Measure for comparison	Correlation hypotheses	Spearman’s Rho
1 Physical condition	SS-QOL sum mobility	High	0.44^a^
	EQ5D mobility	Moderate	0.31^a^
2 Cognitive function	SS-QOL sum thinking	Moderate to high	0.65^a^
3 Emotions	SS-QOL sum mood	High	0.66^a^
	HADS-total score	Moderate to high, negative	−.0.70^a^
4 Daily activities	SS-QOL sum work	Moderate to high	0.62^a^
	EQ5D Usual activities	High	0.64^a^
5 Personal and social life	SS-QOL sum social role	Moderate	0.55^a^
	HADS total score	Moderate, negative	−0.61^a^
6 Current situation and future prospects	EQ VAS score	High	0.57^a^
	HADS anxiety scale	High, negative	−0.58^a^
Sum QOLIBRI-OS	HADS total score	High, negative	−0.74^a^
	EQ VAS score	Moderate	0.56^a^
	SS-QOL sum score	High	0.71^a^

*EQ5D* EuroQol Quality of Life Scale-5D, *HADS* Hospital Anxiety and Depression Scale, *SS-QOL* Stroke-Specific Quality of Life Scale

^a^Correlation is significant at the 0.01 level (two-tailed)

## Statistical and psychometric analyses

The Consensus-based Standards for the Selection of Health Measurement Instruments (COSMIN) guidelines [41] were used as guidelines for this validation study. The psychometric classical test theory analyses were conducted in Mplus version 7.4 [43] whereas all other inferential analyses were conducted in IBM SPSS version 23.

### Descriptive characteristics

The QOLIBRI-OS items were described in terms of means and distributional properties. The degree of floor and ceiling effects, as defined by more than 15% of responses in the extreme lower or upper categories of the scale, were reported [44].

### Uni-dimensionality

A confirmatory factor analysis was conducted to examine the fit of the QOLIBRI-OS as a uni-dimensional model. The maximum likelihood with robust standard errors (MLR) was applied, as the item distributions were non-normal. Model fit was evaluated in terms of the root mean square error of approximation (RMSEA), the standardized root mean square residual (SRMR), the comparative fit index (CFI) and the non-normed fit index (NNFI) [45]. West et al. [45] suggest that RMSEA < 0.05, CFI > 0.95, NNFI > 0.90 and SRMR < 0.06 represent a well-fitting model, while CFI > 0.90, NNFI > 0.85, RMSEA < 0.08, and SRMR < 0.10 indicate a tentatively adequate model.

### Reliability

Cronbach’s α was used to investigate the internal consistency. A value larger than 0.70 is generally recommended for research purposes (e.g., group comparisons), whereas values above 0.90 is desirable for individual clinical assessment [46]. Correlations between QOLIBRI-OS items and its total score were examined (values > 0.40 are preferable) [44] to identify items contributing poorly to the reliability or the ranking of the patients. Test-retest reliability was evaluated with intra-class correlation coefficients (ICCs) based on a two-way mixed model (i.e., treating items and subjects as fixed and random components, respectively). Both ICC absolute agreement and ICC consistency estimates were extracted for comparison purposes [47]. ICC consistency values > 0.75 was considered as excellent.

## Results

### Item characteristics and data quality of the QOLIBRI-OS

The degree of missing QOLIBRI-OS data was below 0.5% (Table 3). Single items were moderately positively skewed. The QOLIBRI-OS total score did not show floor or ceiling effects according to the COSMIN criterion we used, whereas a modest ceiling effect (defined as > 15%) was observed in all items with the exception of one (“Physical condition”). All items robustly contributed to the overall QOLIBRI score, with all item-total correlations above 0.4 (ranging between 0.73–0.88).

### Confirmatory factor analysis (CFA) of the QOLIBRI-OS

The model fit indicators of the hypothesized one-factor model were not universally good (robust χ^2^_df = 9_ = 21.83, *p* = 0.009). Although the relative fit indices were good (CFI = 0.972 and NNFI = 0.953), the important non-centrality index (RMSEA = 0.107) was poorer as opposed to the absolute difference in unexplained standardized residuals that were low (SRMR = 0.029). This model thus yielded mixed support. Removing a single item, i.e., item 3 (“Overall, how satisfied are you with your feelings and emotions?”), yielded a model with excellent universal fit (robust χ^2^_df = 5_ = 3.47, *p* = 0.63; RMSEA = 0; SRMR = 0.015; CFI = 1.0; NNFI = 1.0).

As shown in Table 3, the ICC of the individual QOLIBRI-OS items were high and ranged from 0.75 to 0.91, whereas the overall score had excellent stability, ICC = 0.93.

Internal consistency of the QOLIBRI-OS overall score was excellent (Cronbach’s α = 0.93). We also calculated Cronbach’s α after removing the item “feelings and emotions”.

to observe changes in the internal consistency. In the resulting five-item scale, the Cronbach’s α declined from 0.93 to 0.90.

### Construct validity

As the results of the CFA were mixed, and as the authors considered the item in question (item 3) important for evaluations of HRQOL after stroke, additional correlation analyses were performed. First, the correlation between the five-item (after removing item 3) and six-item overall QOLIBRI-OS was 0.99. Second, the correlations between the HADS, EQ-VAS and the SS-QOL, and the five-and six-item QOLIBRI-OS yielded almost identical results.

**Table 3:** Psychometric properties of the QOLIBRI-OS in 125 participants post-stroke: missing, mean values, item-total correlations and floor and ceiling effects. Test-retest reliability in 36 participants

Item *N* = 125	Missing %	Mean (SD)	Corrected item-total correlation	Floor and ceiling effects (%)	Test-retest reliability *N* = 36
QOL1: Physical condition	0	3.47 (1.02)	0.74	5.6 12.6	0.81
QOL2: Cognitive function	0	3.58 (1.06)	0.73	2.4 20.0	0.87
QOL3: Emotions	0.8	3.58 (1.08)	0.85	3.2 20.8	0.80
QOL4: Daily activities	1.6	3.75 (1.11)	0.78	4.0 28.8	0.91
QOL 5: Personal and, social life	0	3.62 (1.19)	0.83	7.2 24.8	0.75
QOL 6: Current life and, future prospects	0	3.50 (1.09)	0.88	6.4 17.6	0.84
QOLIBRI-OS sum score	0.4	3.58 (0.93)		0.8 7.2	0.93

## Discussion

The results of this study indicated that the QOLIBRI-OS had excellent internal consistency, with slightly higher values than those reported in comparable studies after TBI and subarachnoid haemorrhage [22, 24]. All item-total correlations were high, and the items thus significantly contributed to a reliable ranking of patients. According to the COSMIN guidelines, floor and ceiling effects should not exceed 15% [41]. In our study population, the summary QOLIBRI-OS score had no floor or ceiling effects. Modest ceiling effects were observed for the individual items. Stroke populations are very heterogeneous, thus these ceiling effects are difficult to interpret. For instance, certain subgroups are expected to experience few or no cognitive symptoms [48], therefore, the 20% of persons in this study reporting optimal satisfaction with cognitive functioning (item 2) did not necessarily indicate a problem with the scale, but might rather represent a clinical feature of this population [19]. No other studies have specifically investigated ceiling effects for single items in the QOLIBRI-OS, but von Steinbuchel et al. [22] reported a positive skew for all items indicating positive HRQOL in patients with TBI.

The uni-dimensionality of the QOLOBRI-OS received mixed support, as reported by others [49]. Muehlan et al. [49] identified item 5 (personal and social life) as a potentially problematic item after TBI. In the present study the cause of the mixed fit was related to another item (item 3: feelings and emotions). Removing this item led to an excellent model fit for the resulting five-item QOLIBRI-OS. Nevertheless, we retained all items in the final model because the differences in the validity correlations between the six- versus the five-item versions were negligible. Because this item has not been reported as problematic in other studies, and as the model fit of the six-item QOLIBRI-OS in the present study may be considered as fair, future studies should confirm a problem with this particular item before considering its removal. The problem with item 3 could be related to the translation, which differs slightly from the Norwegian CENTER-TBI version. Norwegian language don’t differentiate between the terms “«feelings” and “«emotion”, hence there was a minor problem in back-translation from Norwegian to English. Therefore a Norwegian replication study containing some changes in wording may be performed, investigating whether the translation of the above mentioned item is inaccurate.

### Validity of the QOLIBRI-OS

Analysis of the a priori hypotheses confirmed construct validity. All a priori hypothesis tests, apart from one hypotheses, showed correlations with the selected other measures in the presumed directions and magnitude (Table 2). The correlation between Physical condition and SS-QOL sum mobility was moderate 0.44, though hypothesised to be high.

The COSMIN criteria indicate that construct validity can be supported if the concurrent correlations with other criterion-related variables are in the magnitude and direction hypothesized or predetermined by the authors. The present results uniformly fulfilled the COSMIN criteria [44]. The lowest correlation was observed between the “satisfaction with physical condition” item and the EQ-5D “mobility” question; this finding is not surprising, as the EQ-5D assesses walking ability in isolation, thus overlooking upper arm function and general health [38]. The highest correlation was observed in a negative relationship between item 3 on the QOLIBRI-OS, “satisfaction with feelings and emotions”, and the HADS total score, which assesses psychological distress [31]; this result is in accordance with previous findings [50]. Emotions contribute substantially to HRQOL, and the high correlation between the QOLIBRI-OS emotion item and mental distress supported maintaining this item, even though the CFA indicated that it might be potentially problematic.

### Score reliability as test-retest stability

The ICC was tested using both consistency and agreement methods. The results were nearly identical, indicating that the subjects provided rather identical responses. The test-retest stability was particularly high for the overall scale (ICC = 0.93), which is higher than in previously published studies (ICC = 0.81) [22]. This may relate to differences in time periods of assessment. In our study, all participants performed test-retest at 12 months, whereas in former studies of QOLIBRI-OS, test-retest was investigated from 3 months to 15 years post stroke.The test-retest stability of all single items were comparable excellent.

Summarized, the psychometric results of the QOLIBRI-OS administered after stroke in this study are comparable or better than the results determined after TBI and subarachnoid haemorrhage [22, 24].

### Can single items be considered individual domains?

The literature is ambiguous about the use of single items [51] to assess HRQOL, as single items are less reliable and valid than sum scores. Nevertheless, other scholars have reported that the reliability of global questions regarding HRQOL might be adequate [52–54].

The EQ-5D [36] has scoring options that include the use of single items. In our study, all of the QOLIBRI-OS items appeared to be uniformly consistent. Means, item-total correlations and test-retest stability varied slightly between items and differed slightly from the results of the total QOLIBRI-OS scale. Moreover, the concurrent validity coefficients of the individual items were high, given the high correlations with criterion-related measures, such as the HADS and SS-QOL. A higher ceiling effect for single items compared to the total score can be expected because of more variation within sum scores. More patients after stroke are expected to have optimal function in one specific aspect assessed by the QOLIBRI-OS, than in all aspects.

### Use of QOLIBRI-OS in patients after stroke?

For clinical and research purposes after stroke there is no single preferred choice of outcome measure yet [4]. We performed a literature search in PubMed from 2014 to 2016 and discovered that the MRS was by far the most commonly used outcome measure in stroke research studies published from 2014 to 2016. However, the MRS does not assess the patients’ subjective perspectives of their health and wellbeing and is unable to differentiate between physical and cognitive sequelae, which is an important argument for including a patient reported outcome measure (PROM).

However, can the QOLIBRI-OS, which is a brief measure, collect substantial information about important HRQOL domains for patients after stroke? In our opinion, the QOLIBRI-OS assesses the major consequences of stroke. Compared to the SIS [55] the QOLIBRI-OS contains one item measuring satisfaction with physical condition but lacks detailed measurements of strength and hand function. The SS-QOL which has 49 items, also includes domains that assess vision and energy [14]. Both the SIS and SS-QOL address communication. The lack of measurement of communication abilities (speaking and understanding) presents, in our opinion, a weakness of the QOLIBRI-OS for use post-stroke. The lack of a specific communication component is likely due to the fact that the instrument was developed only with generalizing overall questions, and the communication aspect was included in the overall item assessing cognition. In addition, motor activity was assumed to be included in the item assessing satisfaction with the physical condition. However, in stroke, communicative and motoric problems are frequent specific problems [56]. Therefore, we suggest that two additional new items should be developed and added to the QOLIBRI-OS- For instance, an item from the QOLIBRI scale regarding satisfaction with language and communicative skills and one item assessing motor function could be included and the scale should then be re-validated in a comprehensive stroke population. For the time being, however, we recommend the use of the QOLIBRI-OS in patients after stroke because it provides a short, reliable and valid index of HRQOL after stroke.

### Strengths and limitations of the study

The strengths of this study are that a major proportion of the unselected stroke population admitted to UNN in 2014 is included. Patients were recruited from stroke units and followed through early rehabilitation, in both hospital and community settings. Of the consenting patients, 78% responded to the main questionnaire, despite the broad inclusion criteria and no exclusion of patients with aphasia or cognitive problems. All patients responded during the same time period post-stroke. The data quality was excellent, and the results were consistent.

A significantly higher portion of non-responders was institutionalized. However, the absolute number of patients with considerable functional deficits post-stroke was low in both groups. A total of 14% of non-responders versus 7% of participants had MRS scores of 4 or 5. This finding may limit the validity of the QOLIBRI-OS in the most severely affected patients post-stroke. Due to Norwegian ethical rules, comparisons between consenters and non-participants are possible for the variables age and gender only, which may limit the representativeness of the results. Furthermore, this study did not evaluate responsiveness to change.

The sample size of 125 patients is less than the first original multinational study of the validity of the QOLIBRI-OS [22], which included 795 patients after TBI and thus provided more substantial statistical evidence of the psychometric data quality. Our study is consistent with the sample sizes from other validation studies of HRQOL measures [18, 57].

## Conclusions

The QOLIBRI-OS is a valid and reliable brief HRQOL measure that is appropriate for application to patients after stroke in research and clinical contexts.

## Abbreviations

COSMIN: Consensus-based Standards for the selection of health Measurement Instruments
HADS: The Hospital anxiety and depression scale
HRQOL: Health related quality of life
MRS: Modified Rankin scale
PROM: Patients reported outcome measure
QOLIBRI: Quality of Life after Brain Injury
QOLIBRI-OS: Quality of Life after Brain Injury – Overall Scale
SIS: Stroke Impact Scale
SS-QOL: Stroke Specific Quality of Life
